# Clinical studies of the Research Committee on Intractable Vasculitides, the Ministry of Health, Labour and Welfare of Japan

**DOI:** 10.1007/s10157-013-0845-1

**Published:** 2013-10-05

**Authors:** Hirofumi Makino, Ken-ei Sada

**Affiliations:** 1 Principal Investigator of the Research Committee on Intractable Vasculitides, The Ministry of Health, Labour and Welfare of Japan, Tokyo, Japan; 2 Secretariat of the Research Committee on Intractable Vasculitides, The Ministry of Health, Labour and Welfare of Japan, Tokyo, Japan; 3 Department of Medicine and Clinical Science, Okayama University Graduate School of Medicine, Dentistry and Pharmaceutical Sciences, 2-5-1 Shikata-cho, Kita-ku, Okayama, 700-8558 Japan

**Keywords:** Antineutrophil cytoplasmic antibody-associated vasculitis, Eosinophilic granulomatosis with polyangiitis, Granulomatosis with polyangiitis, Microscopic polyangiitis

## Abstract

In Japan, the Research Committee on Intractable Vasculitides, supported by the Ministry of Health, Labour and Welfare, has been promoting basic and clinical research on vasculitis since 1972. The present Research Committee on Intractable Vasculitides comprises 4 subcommittees under the direction of a Principal Investigator: Basic and Pathological Research Subcommittee, Clinical Research Subcommittee of Small and Medium-sized Vessel Vasculitis, Clinical Research Subcommittee of Large-sized Vessel Vasculitis, and International Cooperation Research Subcommittee. Since 2008, 9 nationwide clinical studies for vasculitis have been conducted and 8 clinical and basic studies are in progress.

## The Research Committee on Intractable Vasculitides, the Ministry of Health, Labour and Welfare of Japan

**Fig. 1:**
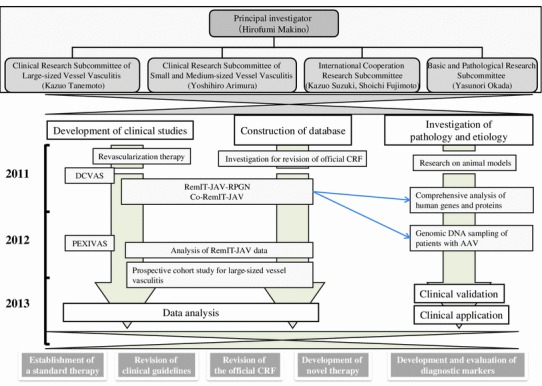
Overview of the tasks of the Research Committee on Intractable Vasculitides. *CRF* case report form, *ANCA* antineutrophil cytoplasmic antibody, *AAV* ANCA-associated vasculitis, *DCVAS* Diagnostic and Classification Criteria in Vasculitis Study, *PEXIVAS* plasma exchange and glucocorticoid dosing in the treatment of ANCA-associated vasculitides, *RemIT-JAV-RPGN* prospective cohort study of remission induction therapy in Japanese patients with ANCA-associated vasculitides and rapidly progressive glomerulonephritis, *Co-RemIT-JAV* observational cohort study of remission maintenance therapy in Japanese patients with ANCA-associated vasculitis, *RemIT-JAV* prospective cohort study of remission induction therapy in Japanese patients with ANCA-associated vasculitides

The Research Committee on Intractable Vasculitides, supported by the Ministry of Health, Labour and Welfare of Japan, has conducted and promoted basic and clinical research on vasculitis since 1972. We study 9 diseases: Takayasu arteritis, temporal arteritis, polyarteritis nodosa, Buerger disease, microscopic polyangiitis, granulomatosis with polyangiitis, eosinophilic granulomatosis with polyangiitis, antiphospholipid syndrome, and rheumatoid vasculitis. Experts from several fields including nephrology, rheumatology, pulmonology, dermatology, cardiology, vascular surgery, pathology, epidemiology, and otorhinolaryngology work cooperatively. The present Research Committee on Intractable Vasculitides comprises 4 subcommittees under the direction of a Principal Investigator (Hirofumi Makino):Basic and Pathological Research Subcommittee of Vasculitis Syndrome (Yasunori Okada), Clinical Research Subcommittee of Small and Medium-sized Vessel Vasculitis Syndrome (Yoshihiro Arimura), Clinical Research Subcommittee of Large-sized Vessel Vasculitis Syndrome (Kazuo Tanemoto), and International Cooperation Research Subcommittee of Vasculitis Syndrome (Kazuo Suzuki, Shoichi Fujimoto) (Fig. 1).

Since 2008, we have conducted a retrospective cohort study elucidating risk factors associated with relapse in microscopic polyangiitis (MPA) patients [1] and a nationwide epidemiologic study of eosinophilic granulomatosis with polyangiitis. The clinical studies described below are in progress currently.

## RemIT-JAV

To describe the current treatment status and evaluate the effectiveness of these treatments for Japanese patients with all types of antineutrophil cytoplasmic antibodies (ANCA)-associated vasculitides (AAV), we conducted a nationwide prospective cohort study of remission induction therapy in Japanese patients with AAV (RemIT-JAV). Twenty-two university hospitals and referring hospitals participated in this study; consecutive patients newly diagnosed with AAV were enrolled from April 2009 to December 2010. The criteria of primary systemic vasculitis proposed by the European Medicines Agency (EMEA) algorithm was employed for enrollment [2]. This study was registered on the University Hospital Medical Information Network Clinical Trials Registry (UMIN000001648). Patients were evaluated at 3, 6, 12, 18, and 24 months and at relapse. The primary outcome measure was remission rate, and secondary outcome measures were survival rate, renal survival rate, and relapse. In total, 156 AAV patients were enrolled; all observations were completed by March 2013. Final data collection is in progress.

## Co-RemIT-JAV

Based on our retrospective study elucidating the risk factors for relapse in patients with myeloperoxidase (MPO)-ANCA positive MPA [1], we are conducting an observational cohort study of remission maintenance therapy in Japanese AAV patients (Co-RemIT-JAV) (UMIN000006373). The study objective is to clarify the safety and efficacy of remission maintenance therapy in Japanese AAV patients. At present, 60 of 156 AAV patients registered in RemIT-JAV were extended to follow up every 6 months up to 48 months after the end of follow-up for RemIT-JAV. The primary outcome measure is relapse rate, and secondary outcome measures are survival and renal survival rates. The observation stage will be completed by March 2015; data collection is currently in progress.

## RemIT-JAV-RPGN

After RemIT-JAV, we conducted a nationwide, prospective cohort study of remission induction therapy in Japanese patients with ANCA-associated vasculitides and rapidly progressive glomerulonephritis (RemIT-JAV-RPGN) (UMIN000005136) including 47 university hospitals and referring hospitals. Enrollment of consecutive patients newly diagnosed with AAV began in April 2011 and will continue till December 2013. The primary and some secondary outcome measures are the same as those in RemIT-JAV, but pathological analysis of renal involvement and radiological analysis of pulmonary involvement will be added. Further, biological samples (serum, urine, and total RNA) will be collected and offered to the Basic and Pathological Research Subcommittee for Research for identifying candidate biomarkers.

## Prospective cohort study for large-sized vessel vasculitis

We also conducted a nationwide Japanese prospective observational study on the current state and efficacy of therapeutics for large-vessel vasculitis (UMIN000010414). The subjects included patients newly diagnosed with Takayasu arteritis and giant cell arteritis. The study objective was to clarify the current state and efficacy of therapeutics for large-vessel vasculitis in Japan and to evaluate the utility of the current diagnostic criteria and classification for large-vessel vasculitis. The primary outcome measure of this study is remission rate. The study began in November 2012, and patients will be registered until March 2014. Final follow-up will be completed in March 2016.

## Other research

The International Cooperation Research Subcommittee is leading the effort to join some international collaborative clinical research studies: the Diagnostic and Classification Criteria in Vasculitis Study (DCVAS) (NCT01066208), the Plasma Exchange and Glucocorticoid Dosing in the Treatment of ANCA-Associated Vasculitis (PEXIVAS) Study (NCT00987389), and a comparison study of phenotype and outcome in microscopic polyangiitis between Europe and Japan.

A genome-wide association study in AAV patients registered in the Japanese clinical studies RemIT-JAV and RemIT-JAV-RPGN, and a prospective study of the severity-based treatment protocol for Japanese patients with MPO-ANCA-associated vasculitis (JMAAV) [3], is also in progress.

## References

[1] Wada T, Hara A, Arimura Y, Sada KE, Makino H (2012). Risk factors associated with relapse in Japanese patients with microscopic polyangiitis. J Rheumatol..

[2] Watts R, Lane S, Hanslik T, Hauser T, Hellmich B, Koldingsnes W (2007). Development and validation of a consensus methodology for the classification of the ANCA-associated vasculitides and polyarteritis nodosa for epidemiological studies. Ann Rheum Dis..

[3] Ozaki S, Atsumi T, Hayashi T, Ishizu A, Kobayashi S, Kumagai S (2012). Severity-based treatment for Japanese patients with MPO-ANCA-associated vasculitis: the JMAAV study. Mod Rheumatol..

